# Evaluation of MEMS NIR Spectrometers for On-Farm Analysis of Raw Milk Composition

**DOI:** 10.3390/foods10112686

**Published:** 2021-11-03

**Authors:** Sanna Uusitalo, José Diaz-Olivares, Juha Sumen, Eero Hietala, Ines Adriaens, Wouter Saeys, Mikko Utriainen, Lilli Frondelius, Matti Pastell, Ben Aernouts

**Affiliations:** 1 Optical Measurements, VTT Technical Research Centre of Finland, 90570 Oulu, Finland; juha.sumen@vtt.fi (J.S.); eero.hietala@vtt.fi (E.H.); 2 Animal and Human Health Engineering, Department of Biosystems, KU Leuven, 2440 Geel, Belgium; jose.diaz@kuleuven.be (J.D.-O.); ines.adriaens@kuleuven.be (I.A.); ben.aernouts@kuleuven.be (B.A.); 3 Mechatronics, Biostatistics and Spectrometers, Department of Biosystems, KU Leuven, 3000 Leuven, Belgium; wouter.saeys@kuleuven.be; 4 MEMS, VTT Technical Research Centre of Finland, 70210 Kuopio, Finland; mikko.utriainen@chipmetrics.com; 5 Natural Resources Institute of Finland Luke, 71750 Maaninka, Finland; lilli.frondelius@luke.fi; 6 Agriculture, Natural Resources Institute Finland Luke, 00790 Helsinki, Finland; matti.pastell@luke.fi

**Keywords:** MEMS, FPI, NIR, milk, analysis, fat, protein, lactose, at-line, on-farm

## Abstract

Today, measurement of raw milk quality and composition relies on Fourier transform infrared spectroscopy to monitor and improve dairy production and cow health. However, these laboratory analyzers are bulky, expensive and can only be used by experts. Moreover, the sample logistics and data transfer delay the information on product quality, and the measures taken to optimize the care and feeding of the cattle render them less suitable for real-time monitoring. An on-farm spectrometer with compact size and affordable cost could bring a solution for this discrepancy. This paper evaluates the performance of microelectromechanical system (MEMS)-based near-infrared (NIR) spectrometers as on-farm milk analyzers. These spectrometers use Fabry–Pérot interferometers for wavelength tuning, giving them the advantage of very compact size and affordable price. This study discusses the ability of MEMS spectrometers to reach the accuracy limits set by the International Committee for Animal Recording (ICAR) for at-line analyzers of the milk content regarding fat, protein and lactose. According to the achieved results, the transmission measurements with the NIRONE 2.5 spectrometer perform best, with an acceptable root mean squared error of prediction (RMSEP = 0.21% *w*/*w*) for the measurement of milk fat and excellent performance (RMSEP ≤ 0.11% *w*/*w*) for protein and lactose. In addition, the transmission measurements using the NIRONE 2.0 module give similar results for fat and lactose (RMSEP of 0.21 and 0.10% *w*/*w* respectively), while the prediction of protein is slightly deteriorated (RMSEP = 0.15% *w*/*w*). These results show that the MEMS spectrometers can reach sufficient prediction accuracy compared to ICAR standard values for at-line and in-line fat, protein and lactose prediction.

## 1. Introduction

The global demand for dairy products increases constantly due to population growth and general elevation of living standards. Controversially, the number of European dairy farms declines and the profitability of farms per animal count stays low. This induces growth in the size of the herds to meet the need and counteract the low profit [1]. Due to large herd sizes, the time of the farmer to attend to each animal is short, which makes it difficult to monitor and manage the animals. Precision livestock farming with on-farm spectrometers is attracting attention as a tool for the automated monitoring of animals with the aim to ensure their health and wellbeing as well as secure production yields and monitor the environmental impact [2]. However, at dairy farms, there is a gap in finding precision livestock tools to achieve accurate on-farm analysis of milk composition. The real-time information on milk ingredients such as fat, protein and lactose would offer the farmer means to balance the diets of individual animals as the cows could receive different supplements, depending on their needs, to further improve the milk output [3]. Moreover, changes in milk quality can indicate alterations in the health and wellbeing of the cows and can influence processed dairy products [4]. In current milk recording practices, the milk quality is monitored at individual cow level only once every 4 to 6 weeks [5]. On-farm PLF tools for milk analysis could provide information on a daily basis and would be a great addition to the official milk recordings. The comparison of the data recorded on-site and analyzed in the laboratory could offer new insights into the periodic changes in cow physiology and wellbeing. The standardized milk testing by accredited central laboratories sets a considerable delay between the moments of taking milk samples and receiving the results. Moreover, as the frequency of sampling is relatively low, there can be changes in cow health reflected by the milk composition that currently go unnoticed. Daily milk recordings have been shown to provide data for continuous health analysis, while the analysis of individual lactations can indicate the cow’s resilience and show how cows differ in their ability to cope with environmental disturbances such as pathogens, heat waves, and changes in feed composition and feed quantity. The automatically collected daily data amounts to such levels that it is feasible to use big data analytics to determine indicators to analyze cow behavior and health [6,7,8,9]. Nevertheless, although several on-farm spectrometers for measuring the milk composition are commercially available [10,11], they are not widely used and their accuracy is often insufficient for monitoring health and welfare and optimizing feed [12,13].

Infrared spectroscopy (IR) is an established method for milk ingredient analysis in central laboratories. Previously, it has shown potential also as a tool for on-farm milk analysis [4,14,15,16]. However, benchtop IR spectrometers are too expensive and bulky to be feasible solutions for on-farm milk analysis. As the profit of the farms is low, the solutions need to be cost-effective [17], practical [18] and easy to implement in current milking stations and robots. Numerous low-cost and compact technologies for NIR wavelength scanning or filtering have been developed in the past years and they have been recently introduced into the market [19]. Several applications on raw milk quality monitoring with miniaturized spectrometers have been recently published [17,18,20,21,22,23], but the reported performances for predicting the milk components were inferior to those reported for benchtop NIR spectrometers and the requirements set by ICAR for on-farm measurements [12].

Miniaturization of NIR detectors for practical on-farm use demands new technological advancements. Microelectromechanical system (MEMS) technology transforms the active components needed for sensors and actuators into a tiny form factor by the use of microfabrication techniques. The components are fabricated using integrated circuit batch processing techniques and their modifications, which enable mass manufacturing and ensure low cost. The development of MEMS has opened up a large variety of affordable sensors for different application fields. The most commonly used sensors include accelerometers, gyroscopes, photodetectors, motion, temperature, image and pressure sensors [24]. In recent years, MEMS-based sensors have been taken into use in agriculture as tools for animal monitoring. They enable the detection of animal behavior and can indicate for example, when an individual cow is in heat [25].

A recent development in the MEMS sensor field is miniaturized spectrographs, which allow the development of portable and handheld spectrometers [26]. Instead of more sophisticated and costly benchtop spectrometers, microspectrometers can be used on-site as they are both practical to use and economical. There are two main methods for spectral scanning in the NIR wavelength range: grating-based scanning with detector arrays [27,28] and interferometric solutions such as Fabry–Perot Interferometers (FPI) [26,29,30]. Compared to grating-based MEMS spectrometers, the FPI scanning offers easy implementation with a high degree of miniaturization [31]. In addition, the fabrication technique of MEMS FPI elements enables mounting them on top of microelectronic devices. This makes the FPI microspectrometers robust and very affordable, thus having a great potential for on-farm milk quality monitoring. This paper studies the ability of miniaturized MEMS NIR spectrometers to analyze the transmittance and reflectance spectra of raw milk in different NIR wavelength ranges and to predict the fat, protein and lactose concentrations. The aim is to evaluate the performance of MEMS FPI NIR spectrometers for on-farm milk analysis and estimate their prediction accuracy as at-line milk composition analyzers.

## 2. Materials and Methods

### 2.1. Milk Analyzer Prototype for MEMS Spectrometer Evaluation

**Figure 1:**
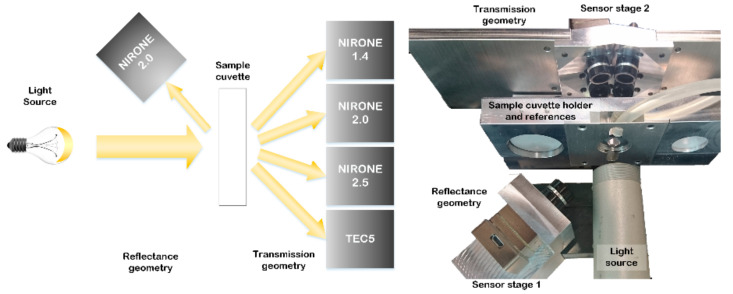
The measurement prototype includes four spectrometers in transmission mode and one spectrometer in reflectance mode as shown in conceptual drawing and picture of the measurement configuration.

The studied MEMS spectrometers utilize small filter components, which consist of thin-film Bragg reflectors with an adjustable short distance cavity between two films. Reflectors function as tensile membranes shifting the air gap and changing the transmitted wavelength [1,2]. These Fabry–Pérot interferometers act as tuneable band-pass filters avoiding the need for movable parts. This enables robust spectrometer performance and offers good long-term stability as well as allows very small size and affordable manufacturing cost [3]. This study evaluated three different spectrometer types including NIRONE 1.4, NIRONE 2.0 and NIRONE 2.5. The original spectrometer designs have been developed by VTT Technical Research Centre [4,5,10] and are currently provided as commercial products by Spectral Engines (Helsinki, Finland) [11]. Each spectrometer type has a customized Bragg reflector design, which determines the scanned wavelength range. The transition distance of the FPI filter membranes sets the wavelength ranges of the spectrometers to be as follows: NIRONE 1.4 reaches from 1100 to 1400 nm, NIRONE 2.0 reaches from 1550 to 1950 nm and NIRONE 2.5 reaches from 2000 to 2450 nm. A benchtop NIR spectrometer (tecSpec PGS 1.7 tc, Tec5, Oberursel, Germany) with a diode array (960–1690 nm) and cooled InGaAs detector, further referred to as TEC5, was used as a benchmark during the experiments. These spectrometers were studied in transmission geometry and an additional NIRONE 2.0 spectrometer was added in reflectance configuration as can be seen in Figure 1.

**Figure 2:**
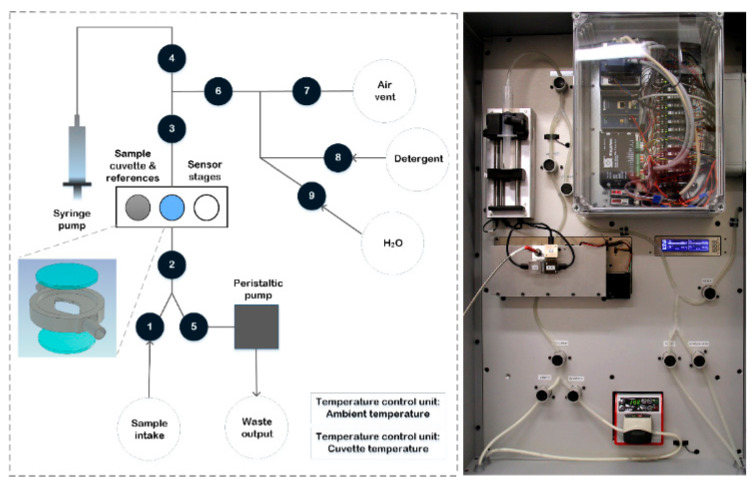
Depiction and picture of the functions of the measurement prototype including pumps, cuvette holder with translation stages, spectrometers and nine valves.

A customized measurement prototype was built to transfer milk samples and record spectral information using the integrated NIR MEMS spectrometers and a TEC5 NIR spectrometer. Each MEMS NIR spectrometer has a price of approximately 1000 €. Picture and depiction of the prototype are presented in Figure 2.

The prototype included a liquid handling system, a temperature stabilization system for the sample cuvette and light source temperature, the spectrometer modules and translation stages. Liquid handling was powered by a syringe pump (New Era NE-500 OEM Application Syringe Pump, AB FIA, Sandby, Sweden) for sample intake and movement and a peristaltic pump (Watson Marlow 120 U, Christian Berner Finland, Vantaa, Finland) for device washing and sample removal. The liquid flow was guided through the measurement cuvette using silicone tubes (OD7ID4, DeLaval, Tumba, Sweden), pinch solenoid valves (S106-08, Sirai, Bussero, Italy) and bubble detectors (BOH0016, Balluff, Neuhausen a. d. F., Germany). The cuvette consisted of a custom-made aluminium body and two quartz windows (Fused Silica UV Grade optically polished 20 mm diameter, 1.5 mm thickness, Crystran Limited, Dorset, UK). The circular cuvette cavity had a diameter of 15 mm and a thickness of 1.5 mm. The cuvette, a white reflectance standard (40% Uncalibrated Spectralon Reflectance Standard, Labsphere, North Sutton, NH, USA) and a white transmittance standard (UV Fused Silica Ground Glass Diffuser, Thorlabs Sweden AB, Göteborg, Sweden) were installed on a translation stage (WGS06K-M57-0250-06 Linear stage, Wexon, Helsinki, Finland), oriented orthogonal to the light beam to bring either the filled cuvette or the spectral standards in the light path. The high-power light source was custom made and incorporated a 65 W bulb and focusing optics. A second translation stage, parallel to the first one, was equipped with a custom-made aluminium light blocker to enable dark standard measurements. White and dark spectral standard measurements were repeated periodically, once every 2 h. The total analysis time of one milk sample, which included the loading of the sample in the cuvette, sample NIR measurements and sample removal, was approximately 90 s.

The recorded light was guided to the MEMS spectrometers and to a TEC5 optical fiber (FG550LEC, Thorlabs, Newton, NJ, USA) using plano-convex lenses (Uncoated, Plano-Convex Lens, Edmund Optics, Barrington, NJ, USA). The lenses were installed at the back side of the cuvette, pointing towards the back center of the cuvette. Moreover, the angle between their axes and the axis of the light source was 165°. The reflected signal was measured by a second NIRONE 2.0 spectrometer with the same plano-convex lenses, pointing towards the front center of the cuvette and with an angle of 45° between its axis and the one of the light source. Although the Fabry–Pérot interferometers enable a compact form factor with a fast signal collection, the detector chips lack cooling and are thus prone to drift more than cooled spectrometers. To reduce this drift and improve the signal-to-noise ratio, each MEMS spectrometer collected 20 subsequent spectra (±10 s) for every milk sample which were averaged afterwards. The benchtop NIR spectrometer measured the transmittance with a single scan and an integration time of 100 ms with 50 scans. The reflectance and transmittance signals of a milk sample were measured simultaneously. After the recording of the NIR spectra, the sample data was automatically corrected using the latest white and dark spectral standard data. The controlling of the pumps, the valves, translation stages, MEMS modules and commercial spectrometer, as well as the correction and storage of the NIR spectra, was directed by a customized software written using LabView 2019 (National Instruments Corporation, Austin, TX, USA) installed on a PC.

### 2.2. On-Farm Milk Analysis

The measurements were conducted at a dairy research farm in Maaninka (Finland), which belongs to the Natural Resources Institute of Finland (Luke). The farm had a herd of 100 cows housed in a free stall with slatted floors and cubicles. Cows had free access to a mixed ration comprising of whole crop silage, wheat and rapeseed concentrate, and a mineral premix. The cows were in different stages of lactation and comprised both Holstein-Friesian and Nordic Red breeds. The milking happened twice a day from 6 a.m. to 8 a.m. and from 3 p.m. to 5 p.m. in a 2-times-8 herringbone milking parlor (SAC, Kolding, Denmark). This study measured samples from three morning and evening milking sessions of three successive days. The measured samples were collected according to the ICAR regulations [12]. Milk was collected into a one liter milk vessel during the milking of one cow. The milk vessel was then gently shaken before the sample was portioned into a reference and a trial sample. A 30 mL reference sample with preservative (±0.3 mg bronopol per ml milk, Broad Spectrum Microtabs II, D and F Control Systems Inc., Dublin, CA, USA) was stored at 4 °C before milk fat, crude protein and lactose analysis were performed by the Valio Oy central laboratory (Seinäjoki, Finland) according to ISO 9622 [13]. The trial sample with 45 mL of raw milk was brought to the room next to the parlour where they were kept at 39 °C for 15 min and stirred gently before analysis with the prototype device. In total, 304 different raw milk samples were collected from 71 individual cows for the measurements. A total of 299 samples were successfully measured without prototype malfunction and had received reference data from the Valio laboratory.

### 2.3. Data Algorithms for Analysis of the Spectra and Prediction of the Milk Composition

The NIR spectra and the respective cow identification numbers and reference composition data (fat, protein and lactose) were imported into R version 3.4.3 [14]. After inspecting the recorded results, three samples were removed from the data set because their spectral files were corrupted, one sample was removed because of missing reference milk composition measurements and one sample was removed because of issues with loading the cuvette just before the automated spectral measurements. Accordingly, 299 milk samples originating from 71 unique cows were kept for further analysis. This data set was then split into a representative calibration set (±2/3rd of samples) and a representative test set (±1/3rd of samples) by applying the duplex algorithm on the autoscaled reference composition data [15,16]. This procedure assured that both sets had similar descriptive statistics. Observations for the same cow were treated as a block with all of them either in the calibration or validation set to prevent overoptimistic validation results in case of modeling cow-specific effects [17]. The set of calibration samples was then used to construct partial least squares regression (PLSR) models to predict the milk components. A separate PLSR model was constructed for every milk component (fat, protein and lactose) in combination with every spectrometer (NIRONE 1.4 transmittance, NIRONE 2.0 transmittance, NIRONE 2.5 transmittance, NIRONE 2.0 reflectance, TEC5 transmittance). The following paragraphs describe the procedure to build a single PLSR model, which was thus repeated for each of the 15 combinations (5 spectrometer × 3 milk components).

Milk NIR spectra are typically subject to light scattering caused by the fat globules and casein micelles. Moreover, the scattering by fat globules can change if the fat globule size changes, even for a fixed fat concentration in the milk [18]. Accordingly, the light scattering typically interferes with the PLSR models and therefore empirical (combinations of) methods are used to filter out the effect of light scattering before constructing the PLSR model. Several combinations of methods to preprocess the NIR spectra were tested in this study. Overall, the process consisted of six consecutive steps, namely the following mathematical treatments (1) a logarithmic spectral transformation (Log) [19] or not (Raw); (2) a baseline correction (Base), detrending (Detr), standard normal variates weighting (SNV) or multiplicative scatter correction (MSC) [20,21,22] or none of those (No process); (3) a first or second (x) order Savitzky–Golay derivative (SGxDyy) with a second-order polynomial filter and 10 different spectral window lengths (yy) [19] or no derivative (No process); (4) either or not an orthogonal signal correction (OSC or No process) with one component to remove the spectral variances which are uncorrelated with the concerned milk component [24]; and (5) mean centering (MNCN). This resulted in 420 different spectral preprocessing techniques, as presented in detail in Aernouts et al. [25]. For each of these 420 combinations, a partial least squares regression (PLSR) model with up to 20 latent variables was built to predict the concerned milk component [26]. Group-wise cross-validation (CV) with 10 groups, each containing spectra of four to five cows randomly selected from the calibration set, was performed on the samples of the calibration set to obtain the root mean squared error of cross-validation (RMSECV). All observations of the same cow were either in the training or the test set of each CV iteration to avoid over-optimistic results when modeling cow-specific effects [17]. We selected the smallest number of latent variables for which the PLSR model was not significantly worse compared to the same model with the number of latent variables resulting in the lowest RMSECV. The statistical comparison in this procedure was based on a one-sided paired *t*-test (α = 0.05) applied on the absolute residuals of the cross-validated observations [27]. A similar approach was followed to select the best spectral preprocessing combination. Moreover, the PLSR models resulting from the 420 combinations were ranked by increasing RMSECV, and the one with the smallest number of latent variables not being significantly worse compared to the model with the lowest RMSECV was selected. Again, a one-sided paired *t*-test (α = 0.05) on the absolute residuals of the cross-validated observations was used to statistically compare the models [25,27]. The milk component of interest is typically only related to the absorption of NIR light at a limited number of wavelengths. Moreover, some regions in the NIR spectra can be irrelevant, while the variation at these wavelengths can confuse the prediction model. This typically results in overfitting with a high number of latent variables and low robustness of the model. Therefore, several variable selection methods were evaluated to see which wavelengths can be removed in order to simplify the regression models and improve their robustness. The selected pre-processing combination (previous section) was applied on the NIR spectra to be used as an input for 4 different variable selection methods: variable importance in projection (VIP), jack-knife (JK), reversed interval partial least squares (RiPLS) and forward interval partial least squares (FiPLS) [28,29,30]. Each of these four methods resulted in a set of most relevant wavelengths for which a PLSR model with an optimal number of latent variables was built as described earlier. The performances of these four PLSR models were compared mutually and with the model that uses all wavelengths. Finally, the set of wavelengths related to the most parsimonious model whose prediction performance was not significantly worse (one-sided paired *t*-test, *α* = 0.05) than that of the model with the lowest RMSECV was selected. Apart from the RMSECV, also the determination coefficient (R²_CV_) was calculated for the samples in the calibration set during cross-validation.

The final prediction model, together with the selected combination of spectral pre-processing methods and the selected set of wavelengths, was used to predict the concerned milk component (either fat, protein or lactose) for all the samples in the validation set based on the respective spectra (either NIRONE 1.4 transmittance, NIRONE 2.0 transmittance, NIRONE 2.5 transmittance, NIRONE 2.0 reflectance or TEC5 transmittance). Based on the retrieved residuals, the root mean square error of prediction (RMSEP) and determination coefficient (R²_P_) were calculated for the entire validation set.

For every milk component, the performances of the different spectrometers were compared using a two-way ANOVA with “spectrometer” as a fixed factor and “sample number” as random factor (=paired test) applied on the squared residuals for each combination of “spectrometer” and “sample number”. If a significant effect (*α* = 0.05) of the “spectrometer” factor was identified, then a Tukey HSD multiple comparison test was performed. This statistical comparison was applied on the cross-validation residuals of the samples in the calibration set first, followed by a similar statistical comparison on the residuals of the samples in the validation set. This statistical comparison of the different spectrometers was repeated for each of the three milk components.

## 3. Results and Discussion

**Figure 3:**
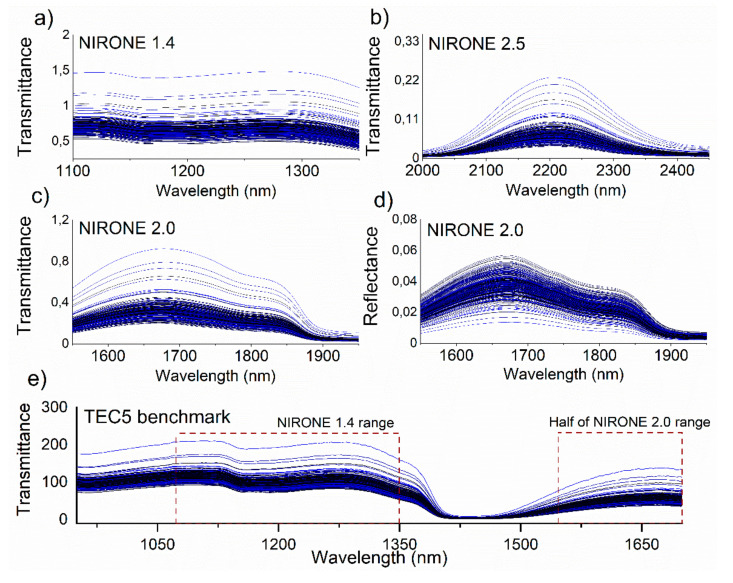
Transmittance and reflectance spectra from Maaninka measurement campaign for (**a**) NIRONE 1.4 spectrometer in transmittance, (**b**) NIRONE 2.5 in transmittance, (**c**) NIRONE 2.0 in transmittance, (**d**) NIRONE 2.0 in reflectance and (**e**) TEC5 benchmark in transmittance with regions overlapping NIRONE ranges indicated.

The performance of MEMS FPI NIR spectrometers for on-farm milk analysis was tested at Maaninka Research farm. During the three-day measurement campaign, a total of 299 milk samples from 71 cows were successfully recorded with reference data from Valio laboratory. Figure 3 shows the spectra (average of 20 scans per sample) of the recorded data for the NIRONE spectrometers as well as the TEC5 benchmark instrument. The corresponding wavelength areas of the NIRONE 1.4 and 2.0 spectrometers have been marked in the TEC5 plot with rectangular boxes. The presented spectra have been corrected for the latest white and dark standard spectra.

The data were first split into calibration and validation groups comprising a set of 205 milk samples (48 cows) for calibration and a set of 94 samples (23 cows) for validation. To ensure that the division represented the original sample group, the basic statistics of the original data group and the calibration and validation groups were compared. The results presented in Table 1 show that the mean, standard deviation, minimum and maximum values of the calibration and validation groups are in the same range as the original sample group. This table also clearly illustrates that the variation in milk fat content is much larger than milk protein, while the variation in milk protein is much larger compared to milk lactose.

In the PLSR calibration, a cross-validation procedure was used to select the optimal number of latent variables of the regression model, the optimal combination of spectral pre-treatment steps and the most favorable variable selection method. The results of the selection process for optimal preprocessing steps and variable selection are presented in Table 2 for each spectrometer and milk component combination.

From Table 2, it can be concluded that there is no preprocessing step or variable selection that clearly performs better for a specific spectrometer or milk component. For reflectance data, it seems that the raw data provide better results compared to the absorbance spectra that are derived through a logarithmic transformation. For transmittance data, both the raw as well as the log-transformation towards absorbance are used in some of the combinations. In the second step, all options except for SNV are applied at some point. The Savitzky–Golay derivative preprocessing is improving the spectral data for most combinations, although the parameters (order and window) patently vary. OSC brings added value to the spectral data for most of the combinations. As variable selection methods, RiPLS and FiPLS are used in nearly all combinations, and they considerably reduce the number of wavelengths that were retained as well as the complexity of the PLSR models. The selected preprocessing steps are in line with other studies [31,32].

**Figure 4:**
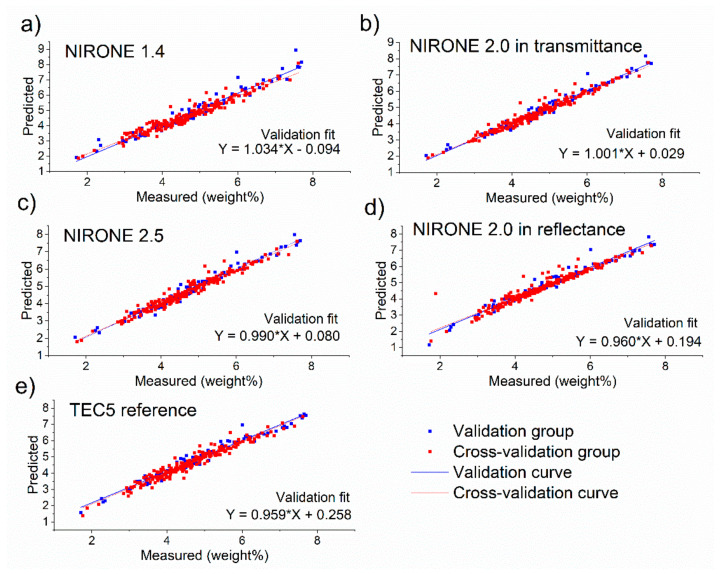
Scatter plots showing the validation and cross-validation predictions compared to measured fat concentration values for (**a**) NIRONE 1.4 spectrometer in transmittance, (**b**) NIRONE 2.0 spectrometer in transmittance, (**c**) NIRONE 2.5 spectrometer in transmittance, (**d**) NIRONE 2.0 spectrometer in reflectance and (**e**) TEC5 spectrometer in transmittance.

**Figure 5:**
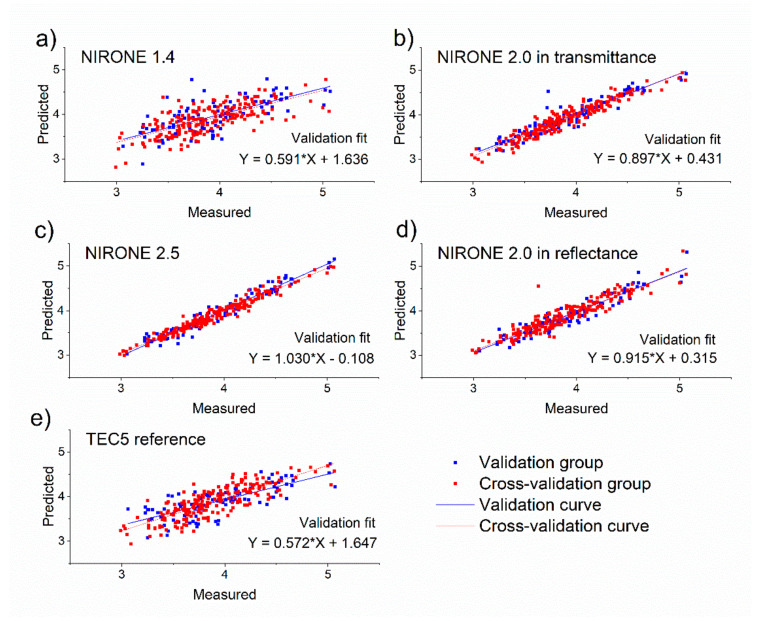
Scatter plots showing the validation and cross-validation predictions compared to measured protein concentration values for (**a**) NIRONE 1.4 spectrometer in transmittance, (**b**) NIRONE 2.0 spectrometer in transmittance, (**c**) NIRONE 2.5 spectrometer in transmittance, (**d**) NIRONE 2.0 spectrometer in reflectance and (**e**) TEC5 spectrometer in transmittance.

**Figure 6:**
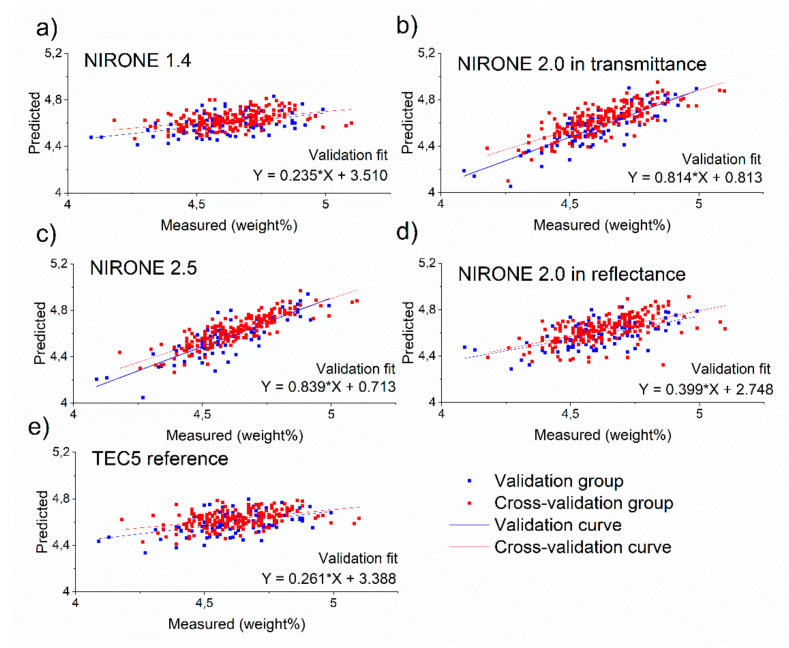
Scatter plots showing the validation and cross-validation predictions compared to measured lactose concentration values for (**a**) NIRONE 1.4 spectrometer in transmittance, (**b**) NIRONE 2.0 spectrometer in transmittance, (**c**) NIRONE 2.5 spectrometer in transmittance, (**d**) NIRONE 2.0 spectrometer in reflectance and (**e**) TEC5 spectrometer in transmittance.

After identifying the spectral preprocessing steps, variable selection and the optimal number of latent variables for the PLSR model, the obtained procedure and PLSR model were applied to the spectral data of the validation set. Accordingly, the retrieved predictions for the 94 samples in the validation set could be compared with the reference analysis in order to obtain the residuals and the RMSEP and R²_P_ values. Apart from the validation set, also the predictions for the 205 samples in the calibration set during cross-validation were obtained, resulting in cross-validation residuals and the derived RMSECV and R²_CV_ values. This procedure was repeated for the 15 combinations of milk components (either fat, protein or lactose) and the spectrometers (either NIRONE 1.4 transmittance, NIRONE 2.0 transmittance, NIRONE 2.5 transmittance, NIRONE 2.0 reflectance or TEC5 transmittance). The results obtained with the best performing model for each spectrometer for the prediction of milk fat, protein and lactose are presented as prediction versus reference scatter plots in Figure 4, Figure 5 and Figure 6, respectively. Figure 4 illustrates a good overall agreement between the predicted and reference milk fat content for all spectrometers tested. There are single points on the scatter plot showing larger deviations from the linear regression line, but this could be attributed to the tendency of fat in raw milk to separate heterogeneously and, despite temperature control and mixing routine, some samples may have been affected by the manual sample intake into the test system. Overall, the results show that the NIRONE MEMS spectrometers can provide comparable results to the TEC5 benchtop spectrometer in fat content determination.

Figure 5 shows the scatter plots for the protein predictions. Here, the subfigures (a–e) indicate that there are clear differences in the prediction performance of the different spectrometers. Moreover, these results indicate that NIRONE 2.5 in transmission, as well as NIRONE 2.0 in transmission and reflectance, are best suited for the protein level predictions, they even outperform the TEC5 benchtop instrument. This is in line with the findings of Aernouts et al., who obtained an accurate prediction of milk protein using transmittance spectra in the 1000 to 2500 nm range or NIR reflectance spectra [32]. In addition, their results confirm that the wavelength range of 1000 to 1700 nm in transmittance is not well suited for protein prediction [32]. This could be the reason why in transmittance mode, the NIRONE 2.0 and 2.5 result in better predictions than the TEC5 benchtop spectrometer with a wavelength range from 960 to 1690 nm.

Figure 6 shows the scatter plots for the lactose predictions. The subfigures (a) to (e) indicate a less cohesive agreement between the predicted and reference concentrations compared to the fat and protein predictions. The best results are obtained in transmittance mode with the NIRONE 2.0 and 2.5 spectrometers. In the study of Aernouts et al. (2011), it was also concluded that transmittance results in superior milk lactose predictions, especially when higher NIR wavelengths were considered [32].

The prediction performance for milk fat, protein and lactose based on the NIR spectra of the five different spectrometers was further evaluated using the number of latent variables of the PLSR models: the RMSECV and RMSEP (Table 3). It is evident from Table 3 that the lowest prediction errors were obtained with NIRONE 2.0 and NIRONE 2.5 spectrometers for all milk components studied. Although the RMSECV and RMSEP values were higher for the NIRONE 2.0 spectrometer in reflectance mode compared to transmittance mode for nearly all milk components, this difference was only significant for lactose. It was reported before that reflectance results in less accurate prediction for lactose compared to transmittance, likely because of less interaction between the reflected NIR light and the lactose molecules in the milk serum [31,32].

For the prediction of milk fat, no significant difference in the RMSECV values between the spectrometers was found. Moreover, all PLSR models included only one or two latent variables, not considering the extra one when OSC preprocessing was used. This illustrates that the PLSR models for milk fat prediction are rather simple and are expected to give a robust performance. Looking at the RMSEP values, only the NIRONE 1.4 transmittance performs significantly worse compared to other NIRONE spectrometers. This might be caused by the fact that it does not overlap with the lower-order fat absorption bands in the higher NIR range that have a stronger relation with the fat concentration. When comparing to other studies using miniaturized NIR spectrometers for raw milk composition analysis, the NIRONE spectrometers resulted in much better milk fat predictions compared to the studies of Yang et al. (RMSECV = 0.43% *w*/*w*) [33] and similar results compared Muñiz et al. (RMSEP = 0.19–0.29% *w*/*w*) [34] and Yang et al. (RMSECV = 0.18–0.33% *w*/*w*) [35]. Still, the milk fat prediction performance of the miniaturized spectrometers was clearly lower compared to benchtop NIR instruments (RMSEP = 0.05–0.09% *w*/*w*) [31,32,36]. This was however not confirmed by the TEC5 NIR benchtop instrument used in this study.

For the prediction of milk protein, the NIRONE 2.0 (both in reflectance and transmittance mode) and NIRONE 2.5 spectrometers performed significantly better compared to the NIRONE 1.4 and TEC5 in transmittance; this is clear both from the RMSECV as well as the RMSEP. Despite its relatively high number of latent variables, the NIRONE 2.5 performed best, although not being significantly better compared to the NIRONE 2.0 spectrometers. The higher complexity of the protein prediction model for the NIRONE 2.5 did not result in overfitting as the RMSEP of this model was clearly the lowest. The protein prediction performances using the spectra of the NIRONE 2.0 (reflectance and transmittance) and 2.5 (transmittance) spectrometers were better compared to the ones obtained with other miniaturized spectrometers used by Yang et al. (RMSECV = 0.34% *w*/*w*) [33], Muñiz et al. (RMSEP = 0.21–0.27% *w*/*w*) [34] and Yang et al. (RMSECV = 0.16–0.23% *w*/*w*) [35].

For lactose prediction, the NIRONE 2.0 and 2.5 in transmittance were significantly better compared to the other spectrometers based on both the RMSECV and RMSEP. Even though their models have a relative high number of latent variables, the lactose prediction performances using the NIRONE 2.0 and 2.5 transmittance spectra seemed robust and were better compared to the ones obtained with other miniaturized spectrometers used by Yang et al. (RMSECV = 0.14% *w*/*w*) [33], Muñiz et al. (RMSEP = 0.13–0.20% *w*/*w*) [34] and Yang et al. (RMSECV = 0.11–0.14% *w*/*w*) [35]. Moreover, they were even close to the performances obtained with benchtop NIR spectrometers (RMSEP = 0.06–0.12% *w*/*w*) [31,32,36].

The accuracy of the prediction models was also analyzed based on the coefficients of determination for the predicted versus reference compositions of the 205 calibration samples in cross-validation (R²_CV_) and the 94 validation samples (R²_P_). Williams has defined the indication limits for approximate quantitative (0.66–0.81), good (0.82–0.90) and excellent (>0.91) prediction [16], which have been used to evaluate the goodness of the calibrations. According to the R^2^ results shown in Table 4, the NIRONE 2.0 and NIRONE 2.5 spectrometers achieve good or excellent fat and protein prediction accuracies. The lactose predictions achieve an approximate quantitative level only with the NIRONE 2.5 spectrometer (R^2^_P_ < 0.67). While the lactose RMSE values meet the ICAR requirements for at-line standards (Table 5), the respective R² values (Table 4) are low because of the limited variability in the lactose concentration of the samples in the calibration and the validation set (Table 1). As the R² values strongly depend on the variation in the data sets, it is important to not only rely on the evaluation of the model performance on these R² values. Apart from the NIRONE 2.5, all other spectrometers, including the TEC5 benchmark spectrometer, showed a very poor performance in terms of the determination coefficient.

The comparison of the prediction errors of the used spectrometers and the ICAR recommendations for on-farm analyzers are presented in Table 5. With an RMSEP below 0.25% *w*/*w* for the prediction of milk fat, all spectrometers except for the NIRONE 1.4 in transmittance mode comply with the requirements set by ICAR for in-line analyzers. Moreover, the RMSEP for the NIRONE 2.0 and 2.5 spectrometers in transmittance of respectively 0.206 and 0.209% *w*/*w* are just slightly too high for at-line analyzers (RMSEP ≤ 0.20% *w*/*w*) [37]. Part of the errors on the fat prediction in this study might result from inhomogeneity due to fat creaming as the NIR measurements were performed 15 min after the milk samples were taken. Although the samples were heated and gently stirred before measuring, this might still have caused a small mismatch between the sample that was analyzed with the experimental setup and the counterpart (reference sample) that was sent out for reference analysis. Automatic splitting and analyzing the sample right after or even during the milking process might reduce this mismatch [31,36] and thus also the prediction error, rendering it compliant with the ICAR requirements.

The protein prediction performances of the NIRONE 2.0 (reflectance and transmittance) and 2.5 (transmittance) spectrometers in this study agree with the conditions set by ICAR for in-line (RMSEP ≤ 0.25% *w*/*w*) and at-line analyzers (RMSEP ≤ 0.20% *w*/*w*) [37]. The milk protein prediction using the NIRONE 2.5 spectrometer in transmittance is even close to the requirements for lab analyzers (RMSEP ≤ 0.10% *w*/*w*) [38].

All spectrometers used in this study meet the requirements for lactose prediction by ICAR for in-line (RMSEP ≤ 0.25% *w*/*w*) and at-line analyzers (RMSEP ≤ 0.20% *w*/*w*) [37], while the NIRONE 2.5 even meets the requirements for lab analyzers (RMSEP ≤ 0.10% *w*/*w*) [38]. The best performing spectrometers were the NIRONE 2.5 and 2.0, which both achieved performance comparable to the ICAR in-line and at-line limits. The NIRONE 1.4 struggled especially with protein and lactose predictions. The TEC5 benchmark instrument covered in comparison a wider wavelength range but did not achieve the same performance level as NIRONE 2.5 and 2.0 during the campaign. This could be related to the missing wavelength range above 1700 nm. However, an earlier study comparing different wavelengths ranges did not observe large differences between 1000 to 1700 nm and 1000 to 2500 nm in transmittance [32] suggesting that the missing wavelength range above 1700 nm in the TEC5 should not play an important role. Although NIRONE 2.0 in reflectance did not achieve as good performance as in transmittance, the results were in a similar range. As the reflectance geometry would be more practical for an in-line detector, it would be interesting in future studies to focus on the optimization of reflectance measurements using both NIRONE 2.0 and 2.5. In addition, it would be important to evaluate the robustness of the calibrations and the spectrometer configurations with more milk samples originating from different farms. This can help to assess the performance level these spectrometers can reach in regards to commercially available systems such as the AfiLab [39,40]. The MEMS NIRONE spectrometers offer the benefits of affordable high-frequency milk analysis [41] as well as the possibility for predictive sensor maintenance and continuous calibrations through the digitalization of farms [42]. This would give the farmers a possibility for accurate and continuous milk analysis offering tools for optimizing individual cow diets and health monitoring.

## 4. Conclusions

This study evaluated the suitability of three MEMS NIR spectrometers, NIRONE 1.4, 2.0 and 2.5, for at-line raw milk analysis at a dairy farm. The Spectral Engines NIRONE 2.5 spectrometer in transmittance mode showed an acceptable accuracy for monitoring the milk fat content (RMSEP = 0.21% *w*/*w*) and an excellent performance (RMSEP ≤ 0.11% *w*/*w*) for protein and lactose. In addition, the transmission measurements using the NIRONE 2.0 spectrometer showed similar results for fat and lactose (RMSEP of 0.21 and 0.10% *w*/*w* respectively), while the prediction of protein was slightly worse (RMSEP = 0.15% *w*/*w*). These results meet the ICAR requirements for at-line milk protein and lactose analysis and nearly reach the conditions for milk fat prediction. In summary, these MEMS spectrometers show promise as future in-line sensors and offer many advantages for the digitalization of dairy farms.

## Figures and Tables

**Table 1:** The descriptive statistics of the (A) original data, (B) calibration data and (C) validation data groups.

Basic Statistics	Milk Composition (% *w*/*w*)								
	Fat			Protein			Lactose		
	A	B	C	A	B	C	A	B	C
Mean	4.715	4.657	4.84	3.899	3.884	3.931	4.62	4.628	4.603
SD	1.107	1.013	1.286	0.415	0.397	0.451	0.154	0.148	0.164
Min	1.71	1.76	1.71	2.99	2.99	3.06	4.09	4.18	4.09
Max	7.7	7.62	7.7	5.07	5.06	5.07	5.1	5.1	4.99

**Table 2:** The optimal preprocessing and variable selection steps identified for each combination of spectrometer and milk component with the following abbreviations: Raw = no logarithmic transformation to absorbance, Log = logarithmic transformation to absorbance, No process = no pre-processing for that particular step, MSC = multiple scatter correction, Base = baseline correction, Detr = detrending; SGxDyy = Savitzky–Golay derivative of order x and with a window yy, OSC = orthogonal signal correction; MNCN = mean-centering, VIP = variable importance in projection, RiPLS = reversed interval partial least squares and FiPLS = forward interval partial least squares.

Spectrometer	Steps	Preprocessing and Variable Selection		
		Fat	Protein	Lactose
NIRONE 1.4 transmittance	1	Log	Log	Log
	2	No process	MSC	Base
	3	SG2D21	SG2D17	SG2D05
	4	OSC	OSC	No process
	5	MNCN	MNCN	MNCN
	6	RiPLS (70WL)	RiPLS (70WL)	FiPLS (12WL)
NIRONE 2.0 transmittance	1	Raw	Raw	Log
	2	Detr	Base	No process
	3	No process	SG2D15	SG1D13
	4	OSC	OSC	OSC
	5	MNCN	MNCN	MNCN
	6	RiPLS (161WL)	RiPLS (142WL)	RiPLS (179WL)
NIRONE 2.5 transmittance	1	Log	Raw	Log
	2	No process	Detr	No process
	3	No process	SG1D21	SG1D19
	4	OSC	No process	No process
	5	MNCN	MNCN	MNCN
	6	FiPLS (45WL)	RiPLS (196WL)	RiPLS (183WL)
NIRONE 2.0 reflectance	1	Raw	Raw	Raw
	2	MSC	No process	No process
	3	SG1D09	SG1D13	SG1D15
	4	No process	OSC	OSC
	5	MNCN	MNCN	MNCN
	6	VIP (66WL)	RiPLS (139WL)	RiPLS (165WL)
TEC5 transmittance	1	Raw	Log	Log
	2	Base	No process	No process
	3	No process	SG1D21	SG2D21
	4	OSC	No process	No process
	5	MNCN	MNCN	MNCN
	6	RiPLS (551WL)	FiPLS (205WL)	RiPLS (165WL)

**Table 3:** The number of used latent variables for the partial least squares regression models obtained for each spectrometer and milk component with respective root mean square error of cross-validation (RMSECV) and prediction (RMSEP) values. Different superscript letters within a column indicate significant (*p* ≤ 0.05) differences according to the Tukey Honestly Significant Difference (HSD) multiple comparison. T = transmittance; R = reflectance.

Spectrometer	Latent Variables			RMSECV (% *w*/*w*)			RMSEP (% *w*/*w*)		
	Fat	Protein	Lactose	Fat	Protein	Lactose	Fat	Protein	Lactose
NIRONE 1.4 T	1	1	1	0.261	0.284 ^c^	0.135 ^b^	0.305 ^b^	0.331 ^b^	0.146 ^b^
NIRONE 2.0 T	1	1	10	0.215	0.121 ^a^	0.094 ^a^	0.206 ^a^	0.153 ^a^	0.101 ^a^
NIRONE 2.5 T	1	11	12	0.21	0.085 ^a^	0.077 ^a^	0.209 ^a^	0.110 ^a^	0.094 ^a^
NIRONE 2.0 R	2	1	7	0.288	0.144 ^a^	0.121 ^b^	0.230 ^a^	0.142 ^a^	0.134 ^b^
TEC5 T	1	11	1	0.234	0.226 ^b^	0.134 ^b^	0.239 ^a,b^	0.295 ^b^	0.147 ^b^

**Table 4:** Coefficients of determination for the partial least squares models of each spectrometer and milk component for cross-validation (R²_CV_) and validation samples (R²_P_). T = transmittance; R = reflectance.

Spectrometer	R²_CV_			R²_P_		
	Fat	Protein	Lactose	Fat	Protein	Lactose
NIRONE 1.4 T	0.933	0.485	0.162	0.943	0.456	0.198
NIRONE 2.0 T	0.955	0.907	0.592	0.974	0.884	0.621
NIRONE 2.5 T	0.957	0.954	0.728	0.973	0.939	0.668
NIRONE 2.0 R	0.919	0.867	0.33	0.968	0.9	0.322
TEC5	0.946	0.675	0.184	0.965	0.568	0.19

**Table 5:** Comparison of spectrometer prediction errors to the ICAR standards.

Spectrometer/Standard	Fat [*w*/*w* %]	Protein [*w*/*w* %]	Lactose [*w*/*w* %]
NIRONE 1.4 T	0.305	0.331	0.146
NIRONE 2.0 T	0.206	0.153	0.101
NIRONE 2.5 T	0.209	0.110	0.094
NIRONE 2.0 R	0.230	0.142	0.134
TEC5	0.239	0.295	0.147
ICAR on-farm in-line standard [37]	0.25	0.25	0.25
ICAR on-farm at-line standard [37]	0.20	0.20	0.20
ICAR laboratory standard [38]	0.10	0.10	0.10

## Data Availability

Data are available in a publicly accessible Zenodo repository: Digital Object Identifier 10.5281/zenodo.5525748.
